# Thermal and Morphological Effect of Low-Tenor Alkali Treatment on Flax and Hemp Fibre Scraps: A Parametric Study

**DOI:** 10.3390/ma19081573

**Published:** 2026-04-14

**Authors:** Sonila Xhafa, Lorenzo Pietracci, Roberto Giacomantonio, Fabio Marchetti, Vincenzo Castorani, Marco Antonini, Roberto Gunnella, Sara Mattiello, Cristiano Fragassa, Carlo Santulli

**Affiliations:** 1 School of Science and Technology, ChIP Research Center, University of Camerino, Via Madonna delle Carceri, 62032 Camerino, Italy; sonila.xhafa@unicam.it (S.X.); lorenzo.pietracci@unicam.it (L.P.); robert.giacomantonio@unicam.it (R.G.); 2 HP Composites S.p.A., Via del Lampo, sn Zona Industriale Campolungo, 63100 Ascoli Piceno, Italy; vincenzo.castorani@gmail.com; 3 Laboratorio. Bioprodotti e Bioprocessi, Ufficio/Laboratorio Camerino, Dipartimento Sostenibilità dei Sistemi Produttivi e Territoriali, ENEA, Via Gentile III da Varano 7, 62032 Camerino, Italy; marco.antonini@enea.it; 4 School of Science and Technology, Physics Division, University of Camerino, Via Madonna delle Carceri, 62032 Camerino, Italy; roberto.gunnella@unicam.it; 5 School of Science and Technology, Geology Division, University of Camerino, Via Gentile III da Varano 7, 62032 Camerino, Italy; sara.mattiello@unicam.it; 6 Department of Industrial Engineering, Università di Bologna, Viale del Risorgimento 2, 40136 Bologna, Italy; cristiano.fragassa@unibo.it

**Keywords:** alkali treatment, hemp fibres, flax fibres, thermal properties, crystallinity, porosity, tensile properties

## Abstract

**Highlights:**

**What are the main findings?**

- In a low-tenor NaOH treatment on hemp and flax scraps, the temperature of the solution appears to be the most significant factor in modifying fibre characteristics by removing loose and worn-out matter.
- The effect on fibre roughness of a higher temperature and immersion time is proven on hemp and less so on flax.
- Calorimetric values on flax indicate a possible modification to the fibre structure at around 250 °C, which might indicate an onset of degradation, which does not appear to be resolved by alkali treatment.

**What are the implications of the main findings?**

- While treatment stiffens both fibres, its effect on the tensile strength of flax, despite the low tenors used, can be considered seriously damaging.
- It appears to be easier to recover hemp than flax scraps for potential use in composites, especially due to their lower proneness to preservation of micro-voids after treatment.
- The possible inclusion of fibre scraps in pellets for thermoplastic processing appears largely conditioned by their different surface characteristics and appears limitedly affected by treatment.

**Abstract:**

The exploitation into new materials of even the smallest scraps of textiles would contribute to their possible success in sectors such as the automotive industry. In this work, alkaline treatment with low sodium hydroxide (NaOH) concentrations was applied to flax and hemp textile residues, aiming to determine the most suitable process conditions as a function of the quality of the treated fibres. Several parameters were considered: the temperature and the concentration of the alkaline solution and the immersion time in the alkaline solution and, eventually, in distilled water during the neutralization phase. Drying tests were carried out under controlled temperature conditions to assess the effects of the various treatment parameters. The effects of the various procedures were elucidated by thermogravimetric analysis (TGA), scanning electron microscopy (SEM), X-ray diffraction (XRD) to assess crystallinity, atomic force microscopy (AFM) to characterize surface roughness, and nitrogen absorption/desorption cycles to determine how microporosity develops with treatment. It is suggested that only the 1.5 wt./vol.% treatment produced some worthwhile modifications of the fibres to prepare them for their use in composites, more evidently in flax than in hemp, though care needs to be taken about fibre embrittlement and potential water permeability.

## 1. Introduction

In the automotive sector, the use of lignocellulosic fibres is being widely investigated for a number of components with various functions in vehicles, typically as the reinforcement for a polymer matrix, for applications such as interior panels, armrests, covers, and other inserts [1]. Several reviews do exist, from over the last two decades at least, to substantiate their interest and effectiveness as renewable materials in car production [2,3,4]. Most recent studies concentrated on singling out which parts of the vehicle can be prospectively manufactured using these materials, in competition with extracted cellulose, thereby adapting them to the purpose by selecting and compatibilizing the fibres as required [5,6,7].

An important drive for this application is that polymer composites reinforced with lignocellulosic fibres are substantially lighter than fibreglass and, in most cases, carbon fibre composites. Beyond that, these fibres represent a renewable filler that can also be employed as a by-product, if not a secondary raw material, from other sectors, such as textiles, thereby providing significant quality at no added cost [8].

Plant fibres from a large number of species have been proposed for automotive applications, such as kenaf [9], pineapple [10], date palm [11], bamboo [12], sisal [13], banana [14], abaca [15], jute [16], nettle [17], etc. In the European context, hemp and flax have proven to be the most viable and available fibres for potential application in the automotive sector [18,19]. A suggestion for the circular economy would be employing fibres that also find application in other sectors, such as, most typically, textiles and construction, and the relevant scraps might therefore offer a good service in parts with cumbersome and irregular sections, even being used as fillers [20,21].

In general terms, to make the fibres more compatible with polymer matrices, surface modification processes are used that reduce the hydrophilicity of the fibres to regularize their geometry [22]. Despite a number of chemical modification processes having been proposed, from an industrial point of view, treatment with an alkaline solution, defined as alkalization or mercerization, is one of the simplest and cheapest methods [23,24,25]. The application of a widely available and easily neutralizable chemical, such as sodium hydroxide, assists in the purification of cellulose in the fibre, removing loose and non-structural material [26,27,28]. In practical terms, this proved to be effective in increasing the amount of alpha-cellulose in treated fibres [29].

Alkaline treatment extracts the secondary components of the fibres, mainly hemicellulose, a very hygroscopic oligosaccharide, and lignin, a polymer composed of a blend of aromatic alcohol monomers. Other components that influence the properties of plant fibres, such as pectin, tannins, and waxes, are present in much smaller proportions and form the amorphous matrix in lignocellulosic fibres, being linked to cellulose via ester and ether bonds as well as via secondary interactions such as hydrogen bonds [30]. During alkaline hydrolysis, these bonds are broken to extract and dissolve the matrix components [31].

Immediately after the alkaline treatment, the fibres must be treated with a bath in distilled water to remove the excess of NaOH from the fibres’ surface [32]. In this way, hygroscopicity is reduced by eliminating very hydrophilic components such as hemicellulose while preventing the hydroxyl groups of cellulose from interacting with water in the external environment. The expected enhancement of the fibres’ mechanical performance can be attributed to the structural realignment of the cellulose chains. Moreover, the elimination of hemicellulose would also improve the compatibility of the fibres’ surfaces with polymeric matrices, thereby increasing the surface roughness of the fibres and improving adhesion and mechanical interlocking between the two materials during the polymerization phase [33]. However, a significant point lies in the fact that excessive alkalinization can produce mechanical damage, especially fibrillation, in the fibres, especially if they are already worn out by previous working, such as is the case for scraps: this will lead to a higher brittleness and possibly induce sudden failure, especially by fibrillation [34].

In this work, different combinations of parameters for the alkaline treatment of scraps from two specific European-origin flax and hemp cultivars were studied. In particular, the factors considered were: (i) the temperature at which the treatment in alkaline solution takes place, (ii) the concentration of the sodium hydroxide (NaOH) solution; and (iii) the immersion time of the fibres in the alkaline solution, which is also related to (iv) the time of subsequent neutralization of the fibres in distilled water. Since these processes will later be used in industry, low NaOH concentrations, not exceeding 1.5% *w*/*v*, were examined to reduce disposal costs for the alkaline broths. The immersion time was minimized as much as possible, aiming to achieve the maximum effect from increasing the solution temperature. Various low-concentration alkaline treatments on hemp and flax fibres were therefore examined, evaluating the mass loss obtained and measuring the thermal properties using thermogravimetric analysis (TGA), comparing the various morphological patterns of the fibres’ surfaces and studying their roughness profile via the analysis of atomic force microscopy (AFM) parameters. The latter methodology provides data suitable to evaluate the potential increase in compatibility of lignocellulosic fibres on polymer matrices due to the effect of chemical treatment [35].

## 2. Materials and Methods

### 2.1. Materials and Treatment

**Figure 1:**
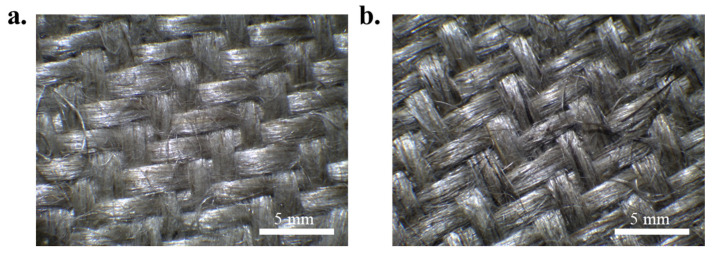
Optical microscopy images (magnification 5×) of (**a**) flax and (**b**) hemp scraps.

HP Composites S.p.A. (Ascoli Piceno, Italy) supplied scraps of untreated plain textile weave materials from hemp and flax fabrics, as residues from production of composites, both with a surface weight of 200 g/m^2^. The fibres were originally obtained from the Futura cultivar in Italy and from the Oliver–Viking crossing cultivar in France, for hemp and flax, respectively (Figure 1).

The flax and hemp fabric scraps were cut into 70 mm side squares, and the resulting samples were oven-dried at 80 °C for 24 h. They were weighed using an analytical balance, and the masses of the samples were in the range between 1.5 and 2 g. Alkali treatment was carried out by immersing each sample in 50 mL of a basic solution in sealed vials. The samples were treated using the process whilst controlling four parameters in detail: the concentration of the alkaline solution, the temperature at which the treatment took place, exposure time to the treatment, and the time of immersion in distilled water. Both fibres were treated with an alkaline solution of NaOH at concentrations of 0.5, 1 and 1.5% *m*/*v*. The solutions were kept at three different temperatures, namely 25, 60 and 80 °C, and different exposure times were carried out for each alkali concentration: 30, 90 and 150 min.

Finally, each treated sample was immersed in a bath of distilled water for 90 min (the water was changed every 30 min) to control the pH, which had to be neutral at the beginning. The fibres were subsequently dried all night at 80 °C in the oven and finally weighed to check the residual mass.

For the alkaline treatments, NaOH tablets with 98% purity and distilled water were used to prepare the alkaline solutions. The fibre treatments were carried out in a ventilated oven at a controlled temperature. To prepare the samples for subsequent characterizations, the fibres were finely pulverized using a Vibromill MV-400., LLC Prizma, Iskitin, Russia).

For comparison, pure microcrystalline cellulose powder (20 micrometres in diameter) was also purchased from Sigma-Aldrich, Vienna, Austria)

### 2.2. Characterization Methods

Infrared spectra of as-received hemp and flax fibres, compared with that obtained from pure microcrystalline cellulose, were recorded from 4000 to 400 cm^−1^ in transmission mode using a Spectrum 100 FT-IR instrument by Perkin Elmer, Milano, Italy)

For the untreated and treated fibres, other characterization methods were also employed as follows.

Thermogravimetric analysis (TGA) was carried out using a STA 6000 simultaneous thermal analyzer by Perkin Elmer, Milano, Italy) under a N_2_ stream (20 mL/min) with a heating rate of 30 °C/min on the raw fibre powder to verify the degree of purification obtained after the alkaline treatment [36].

Scanning electron microscopy (SEM) analysis was carried out using a Scanning Electron Microscope 300 FESEM (field-emission SEM) by Zeiss Sigma, Milano, Italy.

Atomic force microscopy (AFM) images were acquired in tapping mode by using CSI Nano-observer, by Anton Paar, Les Ulis, France, and a P-doped n-type Si cantilever (resonance frequency = 75 kHz). The measurements were performed by using the resonant mode.

The Powder X-ray Diffraction (PXRD) patterns of the treated and untreated fibres were acquired on a D6 PHASER by Bruker Italia, Milano, Italy, equipped with Cu Kα radiation (λ = 1.54060 Å) and a Lynxeye detector, again by Bruker, at 40 kV and 15 mA, with a step size of 0.03° and a step time of 0.05 s, over an angular range of 7–40° 2θ. To determine the crystallinity index (I_c_) of the samples, the powder patterns were deconvoluted by applying a Gauss fitting procedure using the Origin software, version 9.7. The deconvolution process allowed us to identify and separate the crystalline peaks. I_c_ was calculated according to the following Equation (1) from [37]:

(1) $$I_{c}=\left(I_{\left(200\right)}-\frac{I_{\left(am\right)}}{I_{\left(200\right)}}\right)\cdot 100$$

where I_(200)_ represents the intensity peak at around 20–22°, corresponding to the crystallographic plane (200) (crystalline region), and I_(am)_ indicates the intensity peak at around 15.8–16°, lying between the planes (200) and (110) (amorphous region) [38].

The Brunauer–Emmett–Teller (BET) specific surface area and pore size distribution were determined using an ASAP 2020 Micromeritics (Ottawa, ON, Canada) instrument. Nitrogen gas was employed as the adsorbate at −196 °C. Prior to measurements, 2 g of each sample was degassed under a dynamic vacuum at 100 °C until a constant high vacuum was achieved (~10^−5^ bar). BET surface areas were calculated from the N_2_ adsorption isotherms in the 0.01–0.1 P/P^0^ range, adhering to the Rouquerol consistency criteria. Pore size distribution data were also calculated from the N_2_ adsorption isotherms at −196 °C based on nonlocal density functional theory (NL-DFT) model in the Micromeritics ASAP2020 software package.

Differential scanning calorimetry (DSC) analyses were conducted with a Perkin Elmer (Milano, Italy) DSC 6000 with a heating protocol of −20 to 400 °C and a heating rate of 10 °C/min; a nitrogen flow was purged at 30 mL/min. Samples were weighed in the range between 5 and 8 mg, placed into aluminium pans, and then sealed.

Tensile test measurements were performed employing a Shimadzu (Milano, Italy) AGS-X dynamometer equipped with a load cell of 10 kN using a constant speed of 1 mm/min, and the gauge length was fixed at 50 mm. Hemp and flax fibre strands were manually removed from the supplied fabric scraps. Strand fibres were alkali-treated following the same procedure as for the squares. All the fibres obtained were conditioned at a constant temperature of 25 °C and relative humidity of 50% for 48 h before analysis. The diameter of the strand, assumed as circular, was measured with a digital thickness gauge by averaging the measurement in 5 different points of the strand, indicating diameters between 190 and 230 micrometres.

## 3. Results and Discussion

### 3.1. Chemical Groups of Extracted Fibres by FTIR

**Figure 2:**
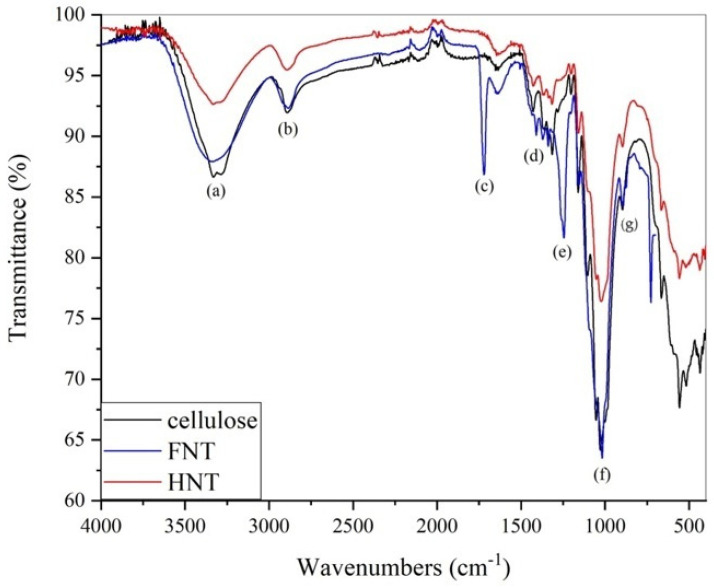
FTIR spectra for the as-received hemp and flax fibres in comparison with pure microcrystalline cellulose.

To initiate the consideration of the results, a brief comment is provided on the chemical properties of the fibres extracted from scraps. In the FT-IR spectra, shown in Figure 2, the characteristic absorption peaks of natural fibres can be observed, as compared with pure cellulose. Going more into detail, the following peaks are observed and reported with gradually decreasing wavenumbers:

(a) An intense broad band is present (3600–3200 cm^−1^), culminating at 3312 cm^−1^, which is due to hydroxyl (-OH) group stretching. These groups are present on the repeated glucopyranoside units, through which intra-chain interactions among the hydrogen atoms are allowed.
(b) Between 2970 and 2840 cm^−1^, a number of lower-intensity absorptions are detected, which correspond to the stretches of aliphatic C-H bonds [39].
(c) A similarly intense band is present at 1712 cm^−1^, associated with non-conjugated carbonyl vibrations, which are suggested to be typical of the presence of ester groups, indicative of the lignin content [40].
(d) Two low-intensity absorptions are present at 1408 and 1338 cm^−1^, associated with bending of C-H and O-H bonds [41].
(e) The band at 1241 cm^−1^ can be assigned to the ethereal groups of aromatic substances, typical of lignin [42].
(f) Very intense absorption peaks are present between 1100 and 1000 cm^−1^, corresponding to C-O-C stretches of acetal groups present in cellulose [43].

The hemp IR spectrum is very similar to pure cellulose, although the absorptions at 1712 cm^−1^ and 1245 cm^−1^ are not present, unlike flax. This evidence suggests the absence of strong covalent interactions, such as ester and ether bonds, between the cellulose and the amorphous matrix. In flax, as reported in [44], the presence of (g) 897 cm^−1^ β-glycosidic linkages between the sugar units in cellulose can also be observed, which are also mostly responsible for the peaks below 700 cm^−1^ [45].

### 3.2. Conditions of Chemical Treatment and Mass Loss

**Figure 3:**
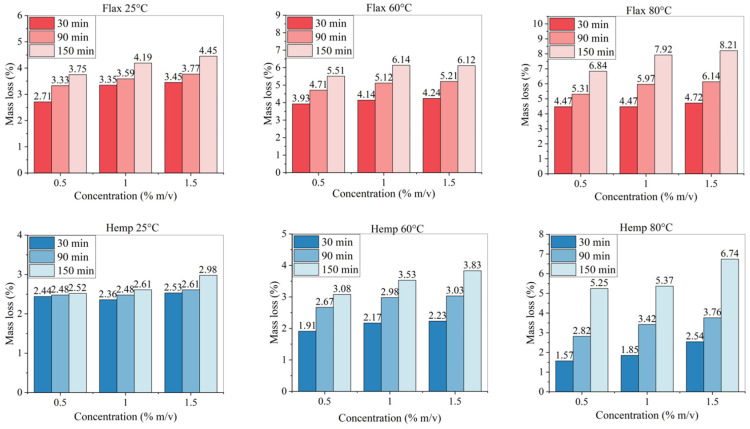
Mass lost (%) for treated fibres with all parameter combinations.

The number of initial procedures investigated for alkali treatment was 27 for each of the two fibres, considering all the combinations of the three parameters (NaOH concentration, immersion time, and solution temperature). The results regarding the mass lost in all circumstances are reported in Figure 3. As the immersion time in the alkaline solution is extended, a larger mass loss is observed, as expected. The parameter that seems to have less influence on the amount of mass extracted is the alkali concentration: increasing it from 0.5 to 1 and 1.5% *m*/*v*, for the same temperature and immersion time, resulted in the mass of the extracted non-structural material not varying significantly across the samples. In flax, the use of a warmer alkali solution increases the mass loss, whereas this is not the case for hemp, unless the maximum immersion time of 150 min is used. This could be explained by the higher amount of non-structural material it contains, namely hemicellulose and other low-molecular-weight polysaccharides. It is also possibly the case that loose and unstructured materials need more time to be dissolved due to the more elongated and variable sectional geometry of hemp fibres [46] with respect to that of flax fibres (more regularly polygonal) [47].

Data on the chemical composition of flax and hemp fibres are largely available, which suggest, from near-infrared (NIR) spectroscopy studies, that, for flax, the amount of cellulose is from 70 up to over 85%, depending on the cultivar [48]: data from previous studies on the Oliver variety indicate a medium-range value of 77% [49]. For hemp, an even larger variation can be found, namely between 55 and over 80% can be found [50]. Post-retting measurements indicated that the Futura hemp cultivar places itself around the maximum possible value for cellulose content [51]. In industrial terms, it appears more practical to try to adapt the same treatment method for both fibres, though it is recognized that its effect might be variable.

Only eight sample categories of the total fifty-four, on which a more detailed study on the effect of treatment was carried out, are reported in Table 1. These were selected by only considering, for temperature, time and NaOH concentration, the minimum (MIN), the medium (MED) and the maximum (MAX) value.

**Figure 4:**
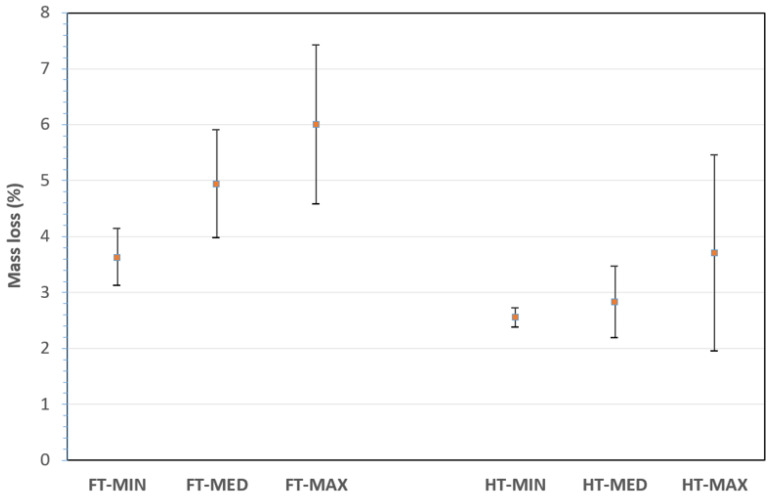
Mass loss (%) for treated fibres with selected combinations.

Figure 4 reports the mass loss after the alkaline treatment for the different sample categories reported in Table 2. The largest mass loss is reported for the FT-MAX category and seems to be mainly due to the combined effect of the warmer temperature and the longer exposition time. This might be expected since the hydrolysis process of the amorphous components of the fibres is strongly endothermic, which is due to the depolymerization of the less-structural cellulose content due to alkali treatment, as also suggested elsewhere [52]. In addition, as already clarified, the treatments remove more matter from flax fibres than from hemp ones. On the other hand, considering the scattering of values, a treatment with a more concentrated and warmer solution for a longer time would be less controllable.

### 3.3. Thermal Properties of Untreated and Treated Fibres

**Figure 5:**
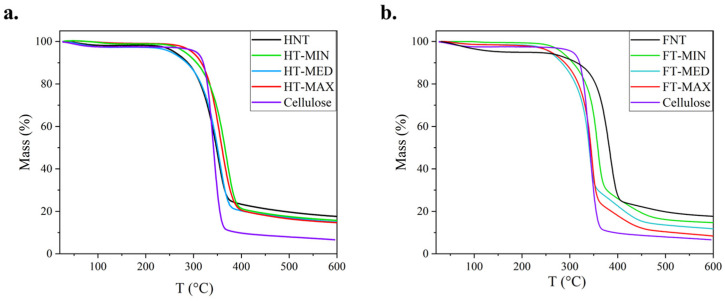
Thermogravimetric analysis (TGA) for (**a**) treated and untreated hemp samples and (**b**) treated and untreated flax samples, in both cases, compared to reference cellulose.

As regards the thermal degradation pattern of fibres, this was assessed using thermogravimetric analysis (TGA), as depicted in Figure 5. This is obviously characterized by the chemical composition of the fibre, as the shape of the curve is influenced by the presence of amorphous components such as hemicellulose and lignin, which affect the tendency of the thermal degradation of the cellulose. The carbonaceous residual mass is mainly constituted by lignin and ash, though it is likely that a limited and progressive degradation of the former would occur in a wide temperature range [53]. This is also due to the high degree of branching and the strong condensation of the aromatic fractions [54,55,56]. The trends of these curves are very similar to each other; therefore, the TGA curves of the three most representative samples of the treated fibres were compared with the degradation curves of the raw fibres and pure cellulose. The TGA analysis shows that the alkaline treatments under different conditions did not cause significant changes in the thermal behaviour of the treated samples compared to the untreated ones and to microcrystalline cellulose. The degradation rate, curve shape and residual mass at 600 °C remain very similar to the reference sample.

However, a closer look at the degradation curve of hemp shows more differences, which are indicated in Table 2: the interval for the thermal decomposition of the structural part of the fibre, which is prevalently formed by crystalline cellulose, is reported, such as going from a degradation start temperature (T_ds_) to a degradation end temperature (T_de_). The degradation range is reconstructed in accordance with the method applied, e.g., in [57], according to the intercepts at the two first sections of the typical S curve obtained in the TGA tests for lignocellulosic fibres. The mass loss in this specific interval grows with increasing treatment with flax, especially for FT-MAX fibres, while this trend is not so consistent for hemp fibres. What is clearly reported is that the residue at 600 °C comes closer to that measured for the pure cellulose, where the degradation pattern is also more rapid and requires less thermal energy.

**Figure 6:**
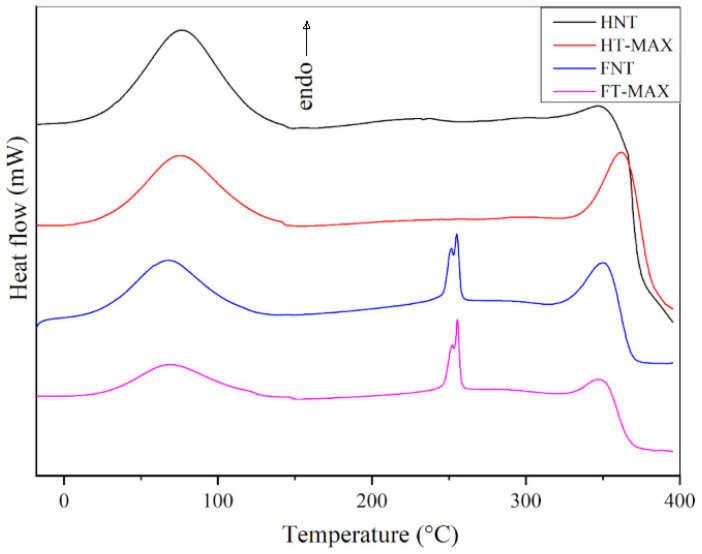
Differential scanning calorimetry (DSC) analysis for max-treated and untreated samples.

DSC curves for the untreated samples and for those subjected to the maximum treatment, as shown in Figure 6, exhibit two main endothermic peaks with notable similarities. The first, a broad peak with a maximum between 67 and 75 °C, corresponds to the release of absorbed water. The second peak, occurring, in every case, not below 315 °C, is associated with the collapse of the cellulose structure [58,59]. This decomposition temperature is slightly higher in the treated samples compared to their untreated counterparts, indicating enhanced thermal stability in both HT-MAX and FT-MAX fibres. The effect is particularly pronounced in the hemp fibres, where the peak shifts from 347 to 362 °C. A slight increase is also observed in the treated flax sample. This improvement may be attributed to increased cellulose crystallinity in the hemp fibres following alkaline treatment, as confirmed later in this paper by XRD analysis, which likely results in a more thermally stable material [60]. More generally, comparing the DSC with the TGA data, it can be suggested that the former indicate that the onset of thermal phenomena is delayed with respect to the beginning of mass loss associated with cellulose degradation. Additionally, a sharp peak around 250 °C is observed in both the FNT and FT-max samples, which may be due to dewaxing or local lignin degradation phenomena [61,62], likely to be due to the cleavage of α- and ß-aryl-alkyl-ether linkages, as reported in [63]. No significant thermal events are detected between 150 and 320 °C for the HNT and HT-MAX samples, indicating their stability within this temperature range.

### 3.4. Crystallinity Measurements by X-Ray Diffraction (XRD)

**Figure 7:**
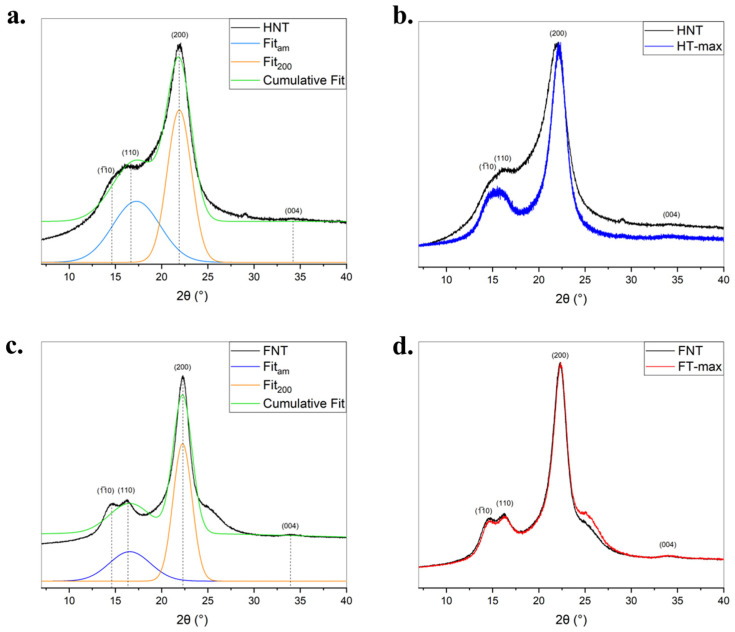
XRD profiles of untreated hemp (**a**) and flax (**c**) fibres (black lines) with their deconvolution curves (crystalline peaks: orange lines; cumulative fit curve: green line); PXRD patterns of the untreated and treated hemp (**b**) and flax (**d**) fibres and *I_c_* (%) values calculated for the fibres.

The structural order and crystallinity of untreated and alkali-treated flax and hemp fibres were investigated by X-ray diffraction. The diffraction profiles are shown in Figure 7, where peak deconvolution was performed to distinguish between crystalline and amorphous contributions. The key peak at approximately 2θ~22° corresponds to the (200) crystalline plane of cellulose I, while the broad background centred around 2θ~18–19° represents the amorphous regions of the fibre matrix. In both flax (FNT) and hemp (HNT), the XRD patterns exhibit a broad and asymmetric shape typical of semi-crystalline lignocellulosic materials. The fitted curves reveal a distinct but modest crystalline peak (200) superimposed on a large amorphous contribution. This reflects the presence of a disordered matrix composed of hemicellulose, lignin, and disorganized cellulose chains, which hinders the full expression of crystalline features. Following NaOH treatment, the XRD patterns of both flax (FT-MAX) and hemp (HT-MAX) show a marked increase in the intensity and sharpness of the (200) reflection. This enhancement is accompanied by a reduction in the amorphous background, indicating the removal of non-crystalline constituents such as hemicellulose, pectin, and residual lignin. The changes suggest a clear increase in the relative crystallinity of the fibres after alkali treatment. While both fibres show similar trends upon treatment, flax (FT-MAX) exhibits a slightly sharper and more defined (200) peak compared to hemp (HT-MAX), indicating that flax may either start with a slightly higher initial crystallinity. Moreover, the crystallinity index was calculated according to Segal’s method, which has also been extensively applied on native cellulose (see, e.g., the review in [64]). This method has been discussed, and limitations have been suggested, especially on the delimitation of the reliability of peak sharpness measurements and, hence, in amorphous subtraction [38]. It remains nonetheless a fast technique to estimate *I*_c_ and allows for comparative measurements. As shown in Figure 7b,d, hemp fibres (HT-MAX) demonstrate a greater relative increase in *I*_c_ compared to flax (FT-MAX) (Figure 7a,c). In general, alkaline treatment leads to a slight enhancement in the crystalline structure of both flax and hemp fibres. While flax exhibits a naturally higher initial crystallinity and treatment does not appear to increase it, hemp undergoes a more substantial reorganization of its internal structure, reflected in a greater increase in the crystallinity index. It has been suggested elsewhere [65] that even higher values of the crystallinity index of treated flax fibres with up to 10% NaOH could be reached, such as up to 78%; however, after exceeding some limit, a transformation from cellulose I to cellulose II is likely to be produced [66]. Crystallinity index values obtained with this method on hemp are usually lower, often not exceeding 60% [67,68]. 

### 3.5. Study of Absorption/Desorption Isotherms to Measure Pore Distribution

**Figure 8:**
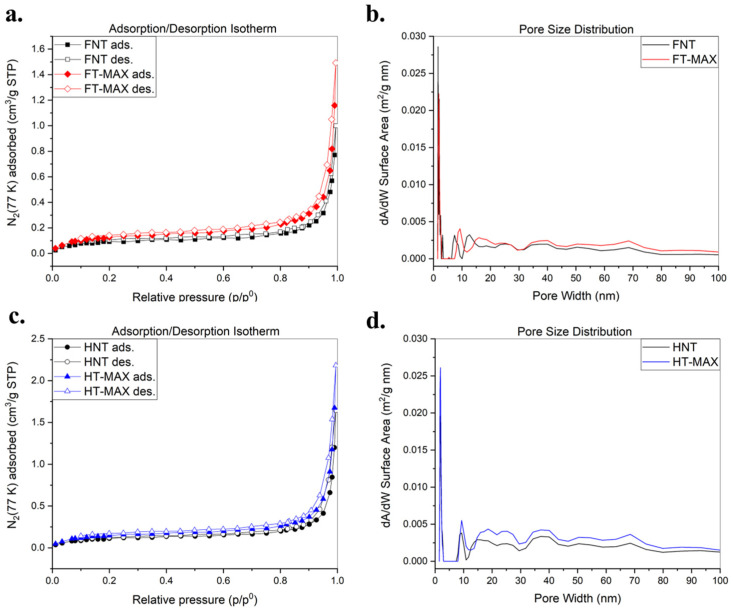
Adsorption/desorption isotherms of treated and untreated flax samples (**a**) and hemp samples (**c**); pore size distribution of treated and untreated flax samples (**b**) and hemp samples (**d**) derived from the N_2_ adsorption data isotherms, calculated by fitting the NL-DFT model.

Figure 8 presents the N_2_ adsorption/desorption isotherms and corresponding pore size distributions for both untreated and alkali-treated fibres to assess their pore distributions using surface area measurements according to the BET theory [69]. As per IUPAC classification, the isotherms generally correspond to Type III, characteristic of non-porous materials. The alkali treatment did not significantly alter the isotherm profiles, although the slight increase in nitrogen uptake, particularly in hemp fibres, suggests a modest improvement in surface area (see Table 3).

The FT-MAX and HT-MAX samples exhibit increased N_2_ adsorption at higher relative pressures in comparison to the initial fibres, indicating enhanced surface area and porosity following treatment. Despite this, the pore size distribution curves remain mostly unchanged, suggesting that while the number and volume of mesopores increased, their size remained consistent. These observations are consistent with known effects of alkaline treatment, which removes surface barriers (e.g., lignin and waxes) and opens up internal voids without drastically altering pore dimensions [70].

### 3.6. Fibre Surface Morphology

**Figure 9:**
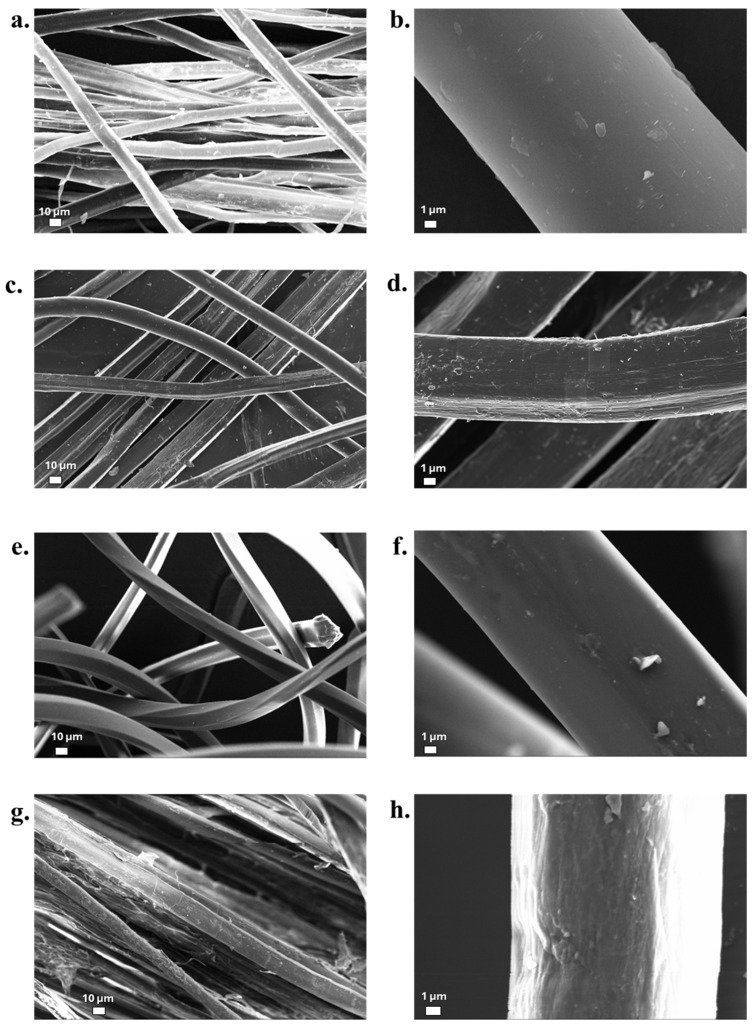
SEM morphological observations for different untreated and treated flax fibres: (**a**,**b**) FNT; (**c**,**d**) FT-MIN; (**e**,**f**) FT-MED; (**g**,**h**) FT-MAX.

**Figure 10:**
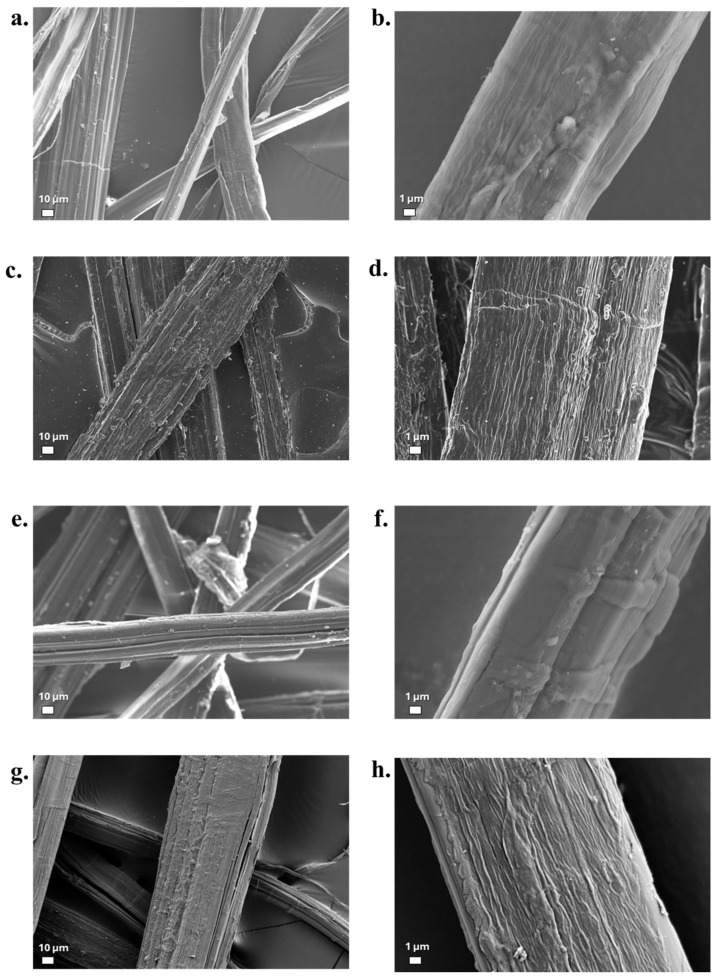
SEM morphological observations for different untreated and treated hemp fibres: (**a**,**b**) HNT, (**c**,**d**) HT-MIN, (**e**,**f**) HT-MED, (**g**,**h**) HT-MAX.

SEM analyses were performed to obtain qualitative information on the morphology of the treated fibres; these are reported in Figure 9 and Figure 10 for flax and hemp, respectively. From the surface morphology of the fibres, it is in fact possible to obtain indications on the adhesion capacity of the fibres to the polymer matrix during the processing phase of composite materials [71,72]. As a general consideration, the surface of the flax fibres appears much smoother, despite the presence of deviated kink bands, which are particularly observed in the untreated fibres and have been widely discussed in the literature [73]. However, the FT-MAX fibres clearly show a much rougher surface and the presence of some loose matter, which might confirm the mass loss data. In the case of hemp, the surface aspect starts from heterogeneous dimensions of individual fibres, and the alkaline treatment with a more concentrated NaOH solution gives greater morphological modification of the fibres’ surface. Other than this, de-fibrillation clearly appears, which is frequently recognized in these fibres, though it does not normally affect their strength [74]. This is especially evident for HT-MAX (Figure 10h).

**Figure 11:**
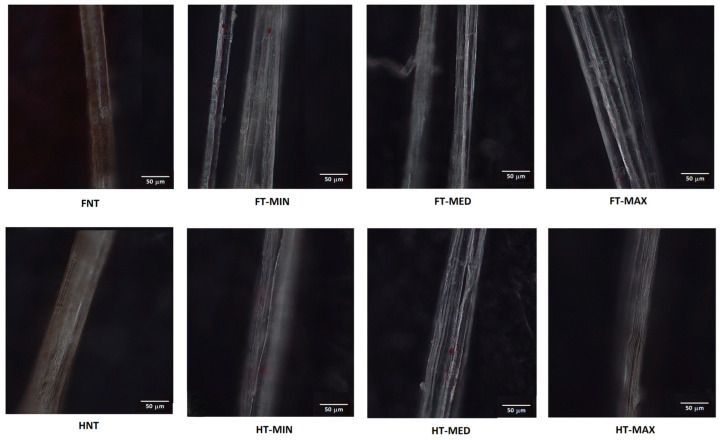
Optical micrographs of samples tested for roughness under atomic force microscopy (AFM).

**Figure 12:**
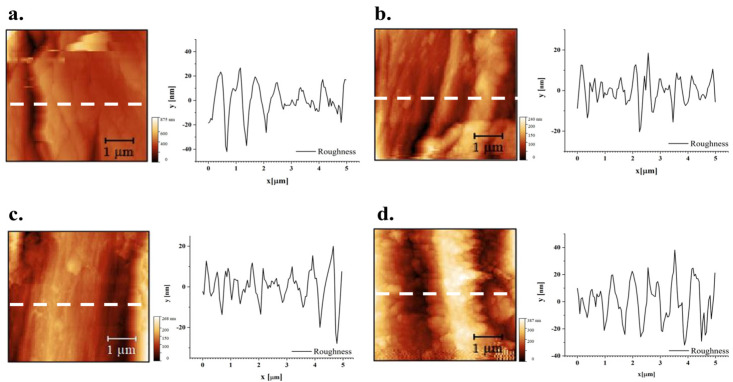
AFM images of flax fibres: (**a**) sample FNT, (**b**) sample FT-MIN; (**c**) sample FT-MED; (**d**) sample FT-MAX.

**Figure 13:**
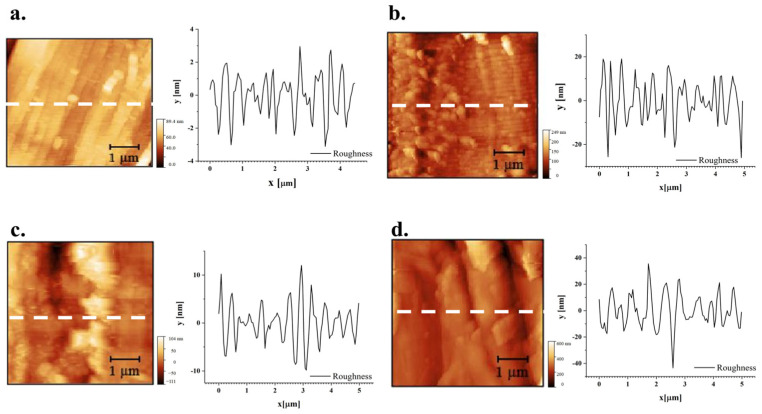
AFM images of hemp fibres: (**a**) sample HNT, (**b**) sample HT-MIN, (**c**) sample HT-MED, (**d**) sample HT-MAX.

In Figure 11, optical micrographs of samples tested for roughness under atomic force microscopy (AFM) are reported. The AFM analyses were particularly concentrated on a single fibre, offering profiles with a length of 5 µm. Figure 12 and Figure 13 show the topography of the flax and hemp fibres, respectively. Previous studies using AFM, namely on flax, documented the extent to which the relevant roughness of the fibre surface can be influenced by the presence of the middle lamella cementing the different fibre cells [75]. This impedes achieving a sufficiently smooth surface in many cases. On the other hand, other AFM observations confirmed the limited dependence of sclerenchyma cells, such as those derived from hemp, on humidity content, practically hindering any swelling process, which also makes roughness measurements more reliable in various environmental conditions [76].

As for the effect of treatment on roughness profiles, depicted in Figure 11 and Figure 12, it is suggested that even the more aggressive conditions tested did not generally modify the surface profiles, possibly only resulting in some reduction in the importance of the tiniest asperities, not exceeding the micron level.

For more thorough considerations, in Table 4, the roughness parameters and values measured are reported. The parameters of the roughness profile are defined as follows: *Rp* = maximum peak height; *Rv* = maximum valley depth; *Rz* = maximum height; *Rt* = total height; *Ra* = average profile; *Rq* = root mean square (RMS) deviation; *Rsk* = skewness; *Rku* = kurtosis. In particular, shifting to the positive value of skewness with treatment, namely on hemp, suggests the more limited presence of surface microvoids that could assist in water penetration [77] and at the same time also affect the achievement of an effective interface in composites [78]. A more refined study would elucidate the repeatability of this outcome and whether treatment can be further tailored for this objective.

### 3.7. Tensile Fibre Measurements

**Figure 14:**
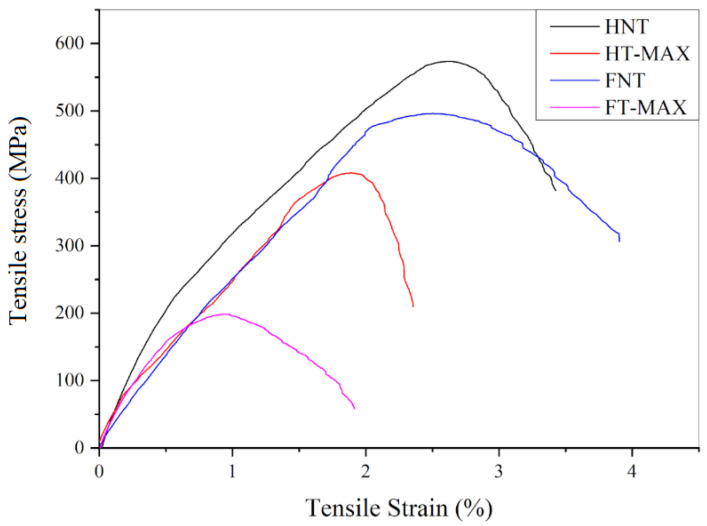
Stress vs. strain representative curves of max-treated and untreated samples.

Figure 14 shows representative stress–strain curves for both treated and untreated fibre bundles. The slight, continuous fluctuations in stress observed during testing were attributed to progressive fracture of individual fibres within the bundle before the final breaking point is reached [79]. The average maximum tensile stress and elongation at break, reported in Table 5, are lower for both hemp and flax fibres after alkali treatment. This decrease can be explained with changes in the chemical composition—particularly the cellulose content—and the internal structure of the two fibre types [80].

**Figure 15:**
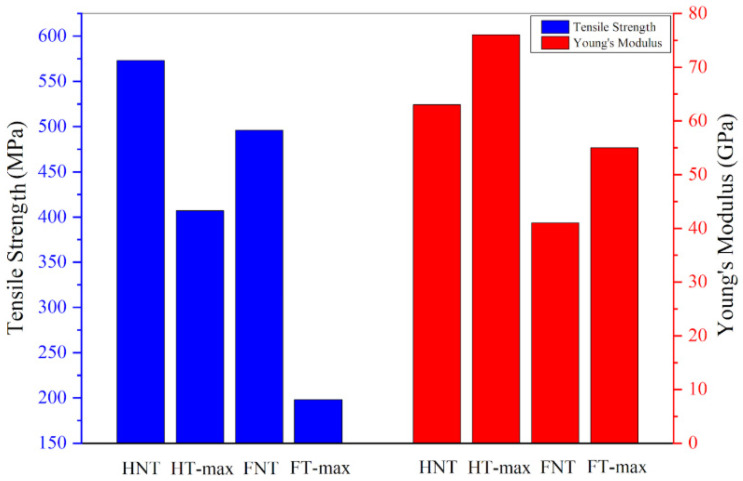
Mechanical properties of max-treated and untreated samples.

As can be seen in Figure 15, the only parameter that clearly increases following alkali treatment is Young’s modulus for both hemp and flax fibres, leading to greater rigidity while possibly increasing brittleness. This increase in stiffness is due to some removal of amorphous components such as hemicellulose, lignin, and surface waxes. Their removal exposes the underlying crystalline cellulose microfibrils, which can better align during mechanical loading, resulting in a stiffer material [81]. In contrast, untreated fibres retain these flexible, non-cellulosic components, which contribute to higher toughness and energy absorption. Without them, treated fibres become more brittle and prone to breaking under lower loads, leading to reduced tensile strength and elongation at break. Additionally, excessive or harsh alkali treatment can damage the cellulose structure or introduce microcracks, further compromising the fibres’ ability to withstand high stress before failure [82]. Values obtained are along the lines of what has been measured in previous classical studies, such as [83] for flax and [84] for hemp, though in the latter case for a crop with a higher crystallinity index, even exceeding 70%.

## 4. Conclusions

In this work, treatments using low-concentration (up to 1.5 wt.%) sodium hydroxide solutions for different immersion times (up to 150 min) and temperatures (up to 80 °C) were studied to verify the set of parameters that would better improve characteristics of flax and hemp fibres. The solution temperature is a parameter that may influence the treatment’s extraction capacity for amorphous components, namely hemicellulose and lignin. Consequently, some changes are found in the characteristics of the treated fibres compared to the starting raw materials, although no major modifications are found upon thermal characterization. The SEM images show very interesting results in terms of morphology, such as evidence that an optimization of the various parameters for NaOH treatment, namely concentration, time of immersion and temperature, can improve fibre surface characteristics. This can also be extended to fibre roughness, where smoothing, more effective in the case of flax, leads to an improved interaction between polymer fibres and matrices, resulting in mechanical interlocking, in the preparation of composite materials. Geometrical modifications raised some concerns about the creation of microvoids by correcting surface profiles and consequent water penetration. Chemical treatment appears to effectively increase the crystallinity of hemp fibres, while the similar effect on flax fibres is limited, if not at all absent, at this tenor of alkali. The only recognized effect that low-concentration alkali can definitely produce is a higher stiffness of both flax and hemp fibres, though at the expense of some brittleness, which was especially noticed on the former, where the strength is more than halved by the maximum level of treatment.

Small differences in NaOH concentration are not expected to produce major effects in the extraction of amorphous components, but the immersion time is also a parameter to take into consideration. Future studies will need to verify whether longer immersion times can improve the efficiency of treatments in order to obtain better materials than those manufactured using untreated fibre scraps. The limitations recognized in this work refer to concentrating on qualifying the relevant parameters for the evaluation of the NaOH treatment effect, also due to the large number of characterization studies performed, rather than performing a complete statistical study.

## Figures and Tables

**Table 1:** Categories of samples examined, identification codes, and relevant treatment parameters.

Sample Category	Temperature (°C)	Time (min)	NaOH Concentration (g/100 mL)
FNT	Untreated		
FNT-MIN	25	30	0.5
FNT-MED	60	90	1
FNT-MAX	80	150	1.5
HNT	Untreated		
HNT-MIN	25	30	0.5
HNT-MED	60	90	1
HNT-MAX	80	150	1.5

**Table 2:** Thermal degradation patterns for the different samples (T_onset_ = onset degradation temperature; T_endset_ = end degradation temperature).

Sample	T_onset_ (°C)	T_endset_ (°C)	Mass Loss Below T_onset_ (%)	Mass Loss Between T_onset_ and T_endset_ (%)	Residue at 600 °C (%)
Cellulose	306	365	5.1	83.7	6.3
FNT	309	408	10.9	61	20.7
FT-MIN	290	370	6.3	62.6	14.7
FT-MED	261	359	5.8	64.8	12.2
FT-MAX	262	391	5.2	76.3	8.3
HNT	273	368	8.3	66.9	19.2
HT-MIN	283	395	5.8	71.9	14.9
HT-MED	272	365	8.1	66.7	13.3
HT-MAX	303	393	6.9	72.6	13.1

**Table 3:** BET surface area of flax and hemp fibres before and after treatment.

Sample Category	δ_BET_ (m^2^ g^−1^)
HNT	0.369 ± 0.01
HT-MAX	0.534 ± 0.011
FNT	0.316 ± 0.008
FT-MAX	0.458 ± 0.01

**Table 4:** Roughness parameters from AFM and relevant values.

Parameter	Flax Samples				Hemp Samples			
	FNT	FT-MIN	FT-MED	FT-MAX	HNT	HT-MIN	HT-MED	HT-MAX
Rp (nm)	26.72	18.49	19.94	38.15	2.95	19.00	12.02	35.50
Rv (nm)	42.02	20.35	27.86	32.02	2.57	26.05	9.84	43.45
Rz (nm)	56.07	28.41	33.01	53.07	4.96	40.06	19.58	50.28
Rt (nm)	68.74	38.84	47.80	70.17	6.06	45.04	21.86	78.95
Ra (nm)	9.92	4.88	5.90	11.91	1.02	7.28	3.03	9.76
Rq (nm)	12.97	6.38	7.79	14.50	1.26	9.08	3.99	12.28
Rsk	−0.60	−0.25	−0.67	−0.04	−0.29	−0.23	0.11	0.04
Rku	3.83	3.89	4.42	2.47	2.75	3.12	3.53	3.73

**Table 5:** Values of mechanical properties and standard deviation, between brackets, for treated and untreated fibres.

Sample	Tensile Strength (MPa)	Strain at Break (%)	Young’s Modulus (GPa)
FNT	496 (150)	3.9 (0.3)	41 (5.8)
FT-MAX	198 (44)	1.9 (0.1)	55 (8.9)
HNT	573 (77)	3.4 (0.6)	63 (9.2)
HT-MAX	407 (149)	2.3 (0.2)	76 (12)

## Data Availability

The original contributions presented in this study are included in the article. Further inquiries can be directed to the corresponding authors.

## Abbreviations

The following abbreviations are used in this manuscript:

AFM	Atomic Force Microscopy
BET	From the Brunauer–Emmet–Teller theory
FTIR	Fourier Transform Infrared Spectroscopy
NIR	Near-Infrared Spectroscopy
NL-DFT	Non-Local Density Functional Theory
SEM	Scanning Electron Microscopy
TGA	Thermogravimetric Analysis
XRD	X-Ray Diffraction
